# Optimal Biomass Allocation to Leaves Across Global Terrestrial Ecosystems

**DOI:** 10.1111/gcb.71166

**Published:** 2026-10-28

**Authors:** Boya Zhou, I. Colin Prentice

**Affiliations:** ^1^ Georgina Mace Centre for the Living Planet, Department of Life Sciences Imperial College London Ascot UK; ^2^ Department of Earth System Science, Ministry of Education Key Laboratory for Earth System Modelling, Institute for Global Change Studies Tsinghua University Beijing China

**Keywords:** biomass partitioning, carbon allocation, climate change, eco‐evolutionary optimality theory, land‐atmosphere exchanges, land‐surface modelling, leaf area index, leaf carbon allocation, leaf economics

## Abstract

Leaves influence global climate and water resources by regulating the flows of CO_2_, energy and water between the atmosphere and land. Here we developed a theoretical framework that predicts the optimal annual fraction of biomass production allocated to the production of new leaves (*η*_f_) as the product of the optimal leaf area index (LAI) and the optimal ratio of leaf mass–per–area to the leaf life cycle (LMA/LC), divided by annual total biomass production (BP). All quantities depend on local environmental variables, with different solutions (embedding a climatic aridity effect on optimal LMA/LC) for evergreen and deciduous plants. Using a global compilation of *η*_f_ data, we show that this theory predicts the emergent responses of *η*_f_ to growing–season temperature and aridity. These responses arise from the trade‐offs among the environmental sensitivities of LMA/LC, LAI and BP. It is predicted that the rising CO_2_ is steadily reducing the fraction of biomass production allocated to leaves.

## Graphical abstract

This study presents an optimality‐based theory that integrates leaf economic traits and the leaf area index–biomass production ratio to predict leaf carbon allocation from environmental conditions. The theory explains global patterns of leaf carbon allocation and predicts a progressive decline in biomass allocated to leaves under rising atmospheric CO_2_.

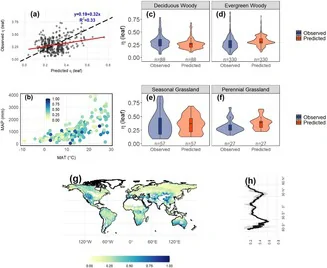

## 1 Introduction

Carbon (C) allocation in plants governs the distribution of photosynthetic products among leaves, stems, roots and reproductive organs, and plays a key role in the function of terrestrial ecosystems (Duanmu et al. 2025; Luo et al. 2003; Poorter et al. 2012). As leaves are plants' main interface for light capture and gas exchange, the allocation of photosynthate to leaves is a major determinant of vegetation productivity, energy balance and biogeochemical feedback on climate change (Zhao and Zhu 2022). Accurate prediction of leaf C allocation is important for understanding and modelling the global C cycle (Zhao and Zhu 2022), and for improving the realism of land surface models that are required for climate forecasting and projection.

However, current vegetation models do not simulate leaf C allocation well (De Kauwe et al. 2014; Franklin et al. 2012). The *fixed ratio* approach, still used in some models, assumes that a constant fraction of net primary production (NPP) is allocated to leaves (Lawrence et al. 2019; Oleson et al. 2010; Wang 2008; Wang et al. 2011). This assumption fails to account for the effects of environmental gradients, including temperature, light and water availability, on leaf C allocation, introducing large uncertainties in global–scale predictions (De Kauwe et al. 2014; Xia et al. 2017). The *allometric biomass partitioning* approach posits invariant scaling relationships between plant tissues (Duanmu et al. 2025; Franklin et al. 2012; Smith et al. 2014; Zaehle and Friend 2010). However, substantial ontogenetic and size–mediated variations in these relationships (Dolezal et al. 2021) challenge this hypothesis. *Resource limitation* theory dynamically adjusts allocation fractions based on growth–limiting resources (Franklin et al. 2012; Meiyappan et al. 2015; Parton et al. 2010); however, when implemented via allocation coefficients (i.e., constant fractions of NPP) this approach creates biases, as shown by its poor performance in forest FACE experiments (De Kauwe et al. 2014).

Models based on optimality principles (Franklin et al. 2012) have usually assumed that allocation coefficients maximise some measure of performance by plants, such as net canopy C uptake. However, by not fully considering the variation of leaf traits, many optimality–based models do not fully capture how leaf C allocation changes with environmental conditions (De Kauwe et al. 2014). For example, leaf mass per area (LMA), a key trait reflecting structural investment in leaves, varies along environmental gradients (Li and Prentice 2024; Wang et al. 2023) – but LMA is assigned a static value (fixed by plant functional type, PFT) in most models, severely limiting their ability to account for dynamic plant–environment interactions (De Kauwe et al. 2014; Lawrence et al. 2019; Oleson et al. 2010; Smith et al. 2014; Wang 2008; Wang et al. 2011; Zaehle and Friend 2010). Canopy leaf area index (LAI) is also poorly represented by many models, often simply as a by–product of a fixed C allocation scheme (Walker et al. 2017) rather than as an optimised quantity that maximises primary production, subject to a trade–off between photosynthetic C gain and transpirational water loss (Cai et al. 2025).

Eco−evolutionary optimality (EEO) theory (Harrison et al. 2021) indicates that C allocation patterns should emerge as a consequence of plants' phenotypic adjustments to environmental constraints via the modulation of plant traits. Therefore, in order to understand the variability of C allocation to leaves, it is necessary to consider both the plasticity of key traits—including leaf lifespan and LMA—and the environmental controls of LAI. A large body of research has established that plants with thicker or denser leaves (i.e., higher LMA) have longer lifespans (i.e., higher leaf lifespan, LL). This relationship is to be expected, reflecting a balance between leaf C gain and the costs of constructing and maintaining leaves (Kikuzawa and Lechowicz 2006; Mediavilla and Escudero 2003; Wright et al. 2004; Ye et al. 2022). Indeed, a proportional relationship between LMA and LL can be shown to maximise average leaf–level net C gain over the leaf life cycle (Kikuzawa 1995; Wang et al. 2023). A prognostic and globally applicable model for LAI dynamics has been developed, based on an optimality principle for maximum LAI (Cai et al. 2025) combined with the concept that leaves are displayed over the period when they are maximally productive (Zhou et al. 2025). These two theoretical approaches, predicting both the global leaf economics spectrum and the amount and seasonal dynamics of LAI, are merged here to provide an EEO–based framework to predict how much assimilated C is allocated to leaves.

Many studies have examined leaf C dynamics based on remotely sensed data. The main focus of this research has been on LAI, neglecting shifts in leaf economic traits, such as LMA and LL (Duanmu et al. 2025; Meng et al. 2023). However, experimental studies found that leaf C allocation is relatively conserved across environmental gradients (Lu et al. 2025; Malhi et al. 2011, 2017; Poorter et al. 2012), despite substantial variation in LAI (Cai et al. 2025). This is possibly because plants employ distinct morphological adaptations to environmental stressors. In dry environments, for example, vegetation typically invests less in leaf area (to reduce water loss) while simultaneously allocating more C to leaf thickening, reflected in higher LMA through increased palisade parenchyma development and cuticular wax deposition (Fletcher et al. 2018; Lawal et al. 2022).

Thus, we hypothesized that the relatively stable fraction of biomass allocated to leaves arises from the coordinated adjustment of leaf area and leaf economic traits relative to total plant biomass production (BP). In our framework, annual leaf biomass production is determined by the amount of leaf area deployed (LAI) and the carbon investment required to construct and maintain leaves, which is represented by LMA relative to leaf lifespan (LMA/LL), whereas BP represents the total annual biomass available for allocation among plant organs. Consequently, the annual fraction of biomass allocated to new leaves (*η*_f_) can be expressed as the product of LAI and LMA/LL divided by BP. This formulation implies that leaf C allocation can remain relatively stable despite substantial variation in LAI if changes in leaf economic traits and total biomass production compensate for changes in leaf area.

Based on this hypothesis and EEO theory, we developed a predictive framework by embedding two recently developed theories for leaf economics (Wang et al. 2023) and leaf area dynamics (Cai et al. 2025; Xin et al. 2020; Zhou et al. 2025) into a first–principles EEO–based model for gross primary production (GPP) (the P model, Stocker et al. 2020; Wang et al. 2017). Then, based on the model predictions and multi–source observational data, we explored the global pattern of leaf C allocation and its governing mechanisms. Finally, we explored the changing trend of modelled leaf C allocation over the past 120 years under the influence of changes in climate and atmospheric CO_2_ and identified the main driving factors behind it.

## 2 Results

### 2.1 Evaluation of Leaf Economics Predictions

Our analysis uses the theoretical model of Wang et al. (2023), which predicts the ratio of LMA to the leaf life cycle (LC) based on the EEO criterion that leaves maximise the return on C investment. LC is equal to 1 year for deciduous plants and to LL for evergreen plants. After accounting for water limitation effects on leaf economics (Wang et al. 2023) (see Methods; Figure S2), the improved LMA model captures the variations of the observed LMA/LC ratio (Figure 1) for all plants together (*R* ^2^ = 0.50) and for different phenological types separately (*R* ^2^ = 0.53 for deciduous and 0.36 for evergreen plants) (Figure S3). Treating site as a random effect does not materially alter these relationships (Figure S3). Furthermore, in dense forests, LMA/LC is observed to decline due to reduced light availability. The improved LMA model successfully reproduces this decline in LMA/LC in densely forested regions with higher LAI_max_ (Figure S4).

**FIGURE 1:**
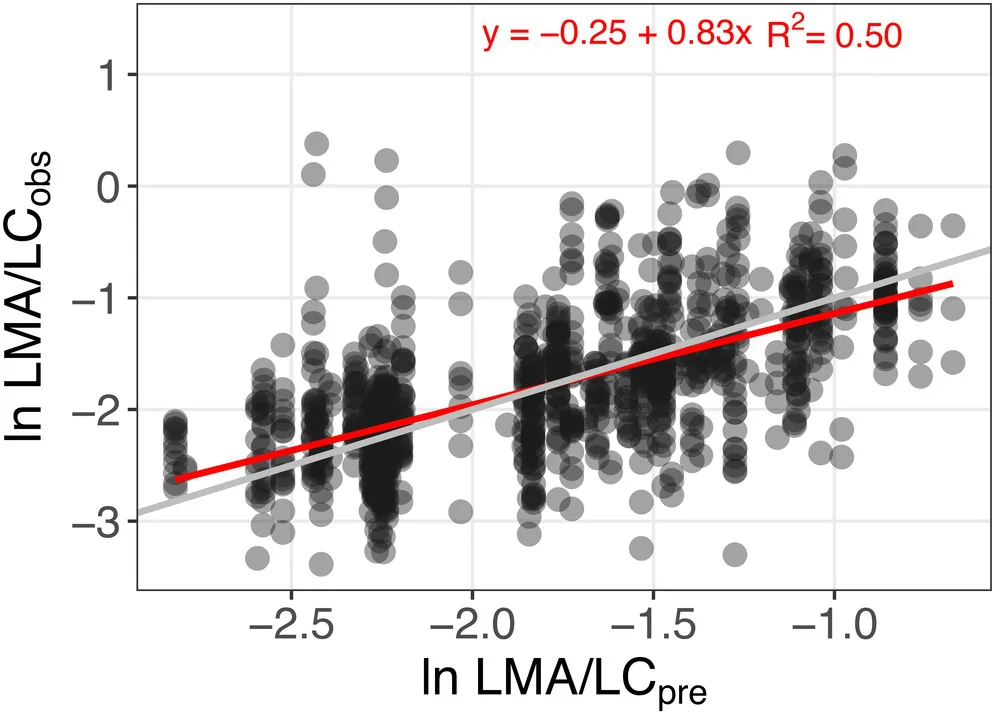
Observed (obs) versus predicted (pre) ratios of Leaf Mass per Area (LMA) to Leaf Lifecycle (LC), where LC is equal to 365 days for deciduous species and to leaf lifespan for evergreen species. The red line is the ordinary least–squares regression line, and the grey line is the 1:1 line.

### 2.2 Comparisons With In Situ Data and Predicted Spatial Patterns

The model broadly reproduces the observed patterns of leaf carbon allocation across woody and herbaceous plants (Figure 2c–f) but explains only 33% of the overall variance in observed *η*_f_ (Figure 2a), indicating that some variation within woody and non–woody groups remains unexplained. Compared with woody plants (32% ± 10% for deciduous and 27% ± 8% for evergreen woody plants), the proportion of biomass production allocated to leaves in herbaceous plants is both larger and more variable (36% ± 19% in seasonally dormant and 31% ± 7% in persistent perennial non–woody plants) (Figure 2c–f). The greater variability among herbaceous plants may reflect sampling limitations and/or greater physiological plasticity in response to environmental stress, particularly drought. The model reproduces the broad differences among these vegetation groups, predicting 26% ± 6% for deciduous and 32% ± 4% for evergreen woody plants, and 35% ± 14% for seasonally dormant and 37% ± 7% for persistent perennial non–woody plants.

**FIGURE 2:**
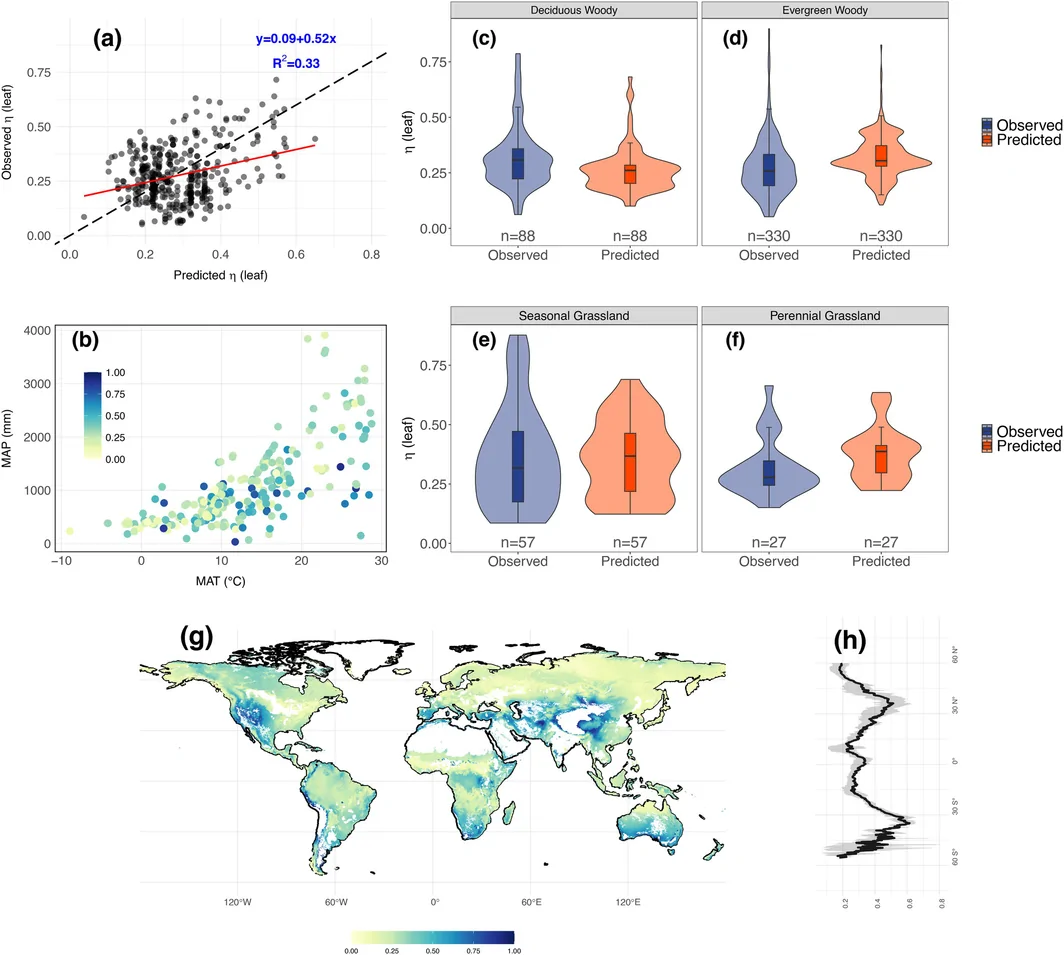
(a) The robust regression between observed and predicted annual fraction of biomass production allocated to the production of new leaves, *η*(leaf). (b) Observed *η*(leaf) in relation to mean annual temperature (MAT) and precipitation (MAP). Predicted (red) and observed (blue) distributions of the fraction of biomass production allocated to leaves for (c) deciduous woody, (d) evergreen woody, (e) seasonally dormant non–woody species and (f) persistent perennial non–woody species. (g) The geographic pattern of predicted mean *η*(leaf) for 1982–2010 and (h) its variation with latitude.

The predicted global average leaf C allocation during 1982–2010 is 0.28 (Figure 2g). The model predicts a larger fraction of biomass production allocated to leaves in drier regions, including western North America, the Qinghai–Tibet Plateau and interior Australia. Alternative data sources for the ratio of seasonally senescent versus persistent perennial plants have minimal impact on these predictions (Figure S9). The global predicted leaf C allocation pattern predicted by the prognostic LAI model (Cai et al. 2025; Zhou et al. 2025) (Figure S9a,c) is similar to that based on MODIS LAI data (Li et al. 2023) (Figure S9b,d).

### 2.3 Variation of Leaf Carbon Allocation

Observed variations in leaf C allocation can be statistically related to mean growing–season temperature (Gst, °C) and Aridity Index (AI) (Figure 3a–d; Table 1). We found no significant effect of stand age (Table S2), possibly due to the uncertainties of available stand–age data (Poulter et al. 2018). Leaf C allocation emerges as a conservative trait, displaying detectable yet small variation in response to environmental shifts (Figure 3a–d) owing to opposing regulatory mechanisms along three axes: leaf economics (quantified by LMA/LC), leaf area dynamics (LAI) and plant total biomass production (BP) (Figure 3e–h; Figure S7).

**FIGURE 3:**
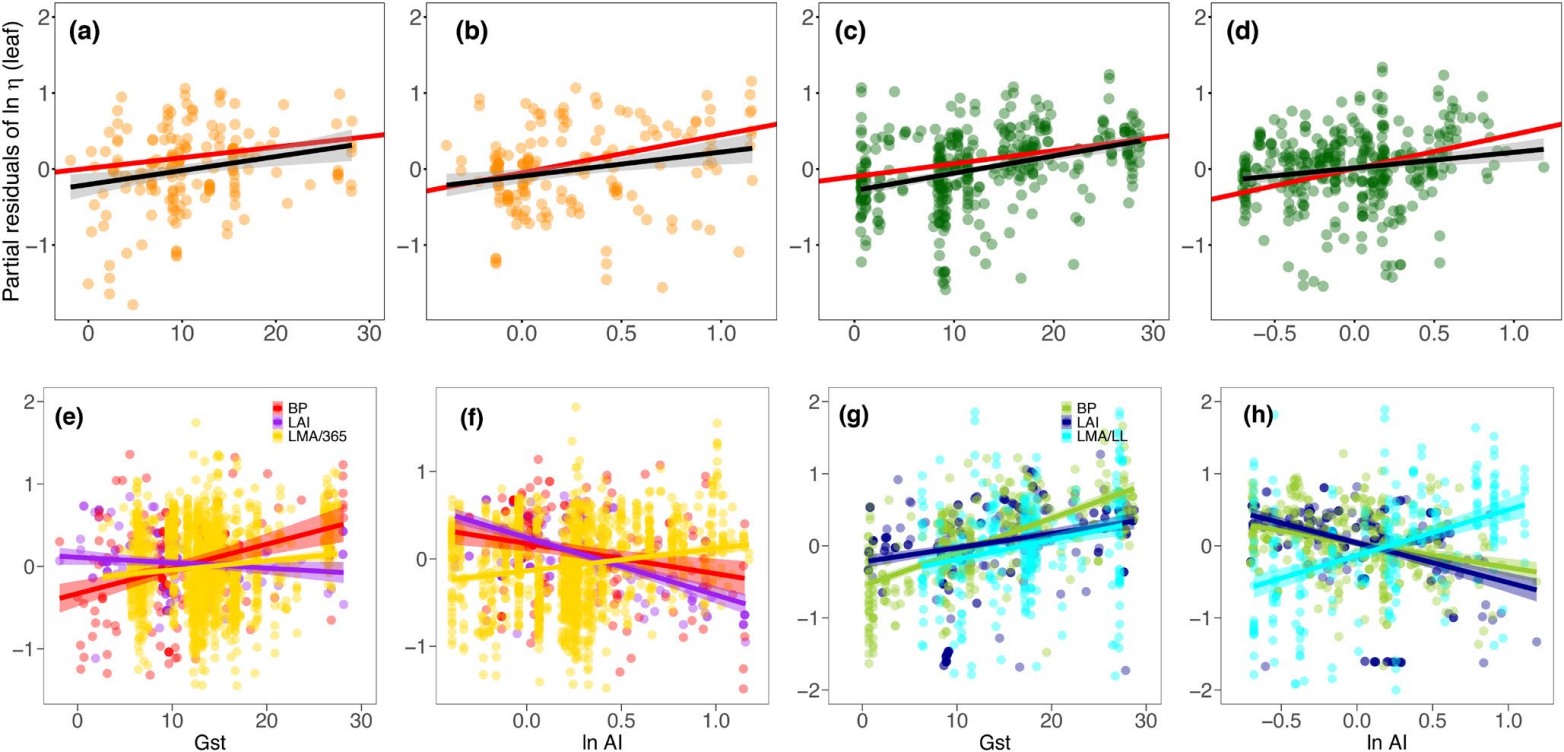
(a–d) Partial residual plots for observed leaf carbon allocation against environmental variables. (a, b) Seasonally senescent plants; (c, d) Persistent perennial plants. Predicted: Red. Fitted: Black, with 95% confidence intervals in grey. (e–h) Partial residual plots for the observed seasonal maximum Leaf Area Index (LAI), observed plant total biomass production (BP), and the observed ratio of Leaf Mass Area (LMA) to leaf life cycle (LC), against environmental variables. Data on observed leaf carbon allocation and plant total biomass production are from Peng et al. (2023) and Lu et al. (2025). Observed LMA/LC ratios are from the China Plant Trait Database (Wang, Harrison, et al. 2022) and Glopnet (Wright et al. 2004). AI, Aridity Index; Gst, mean growing season temperature.

**TABLE 1:** Summary statistics for the regression of observed and predicted leaf carbon allocation against bioclimatic variables, calculated separately for seasonally senescent and LMA has been shown to be closely linked to leaf water content persistent perennial plants.

		Gst	*p*	ln AI	*p*	*R* ^2^	*n*
Seasonally senescent plants	Observed	0.020 ± 0.010	< 0.05	0.400 ± 0.120	< 0.001	0.11	122
Persistent perennial plants		0.020 ± 0.002	< 0.001	0.170 ± 0.050	< 0.001	0.17	343
Seasonally senescent plants	Predicted	0.014 ± 0.007	0.051	0.840 ± 0.080	< 0.001	0.52	122
Persistent perennial plants		0.016 ± 0.001	< 0.001	0.510 ± 0.030	< 0.001	0.66	343

Abbreviations: AI, Aridity Index; Gst, mean growing–season temperature.

Plants allocate a greater *η*_f_ under higher growing–season temperatures and greater aridity (Table 1; Figure 3a–d; Table S3). These increases are driven by coordinated changes in LAI, LMA/LC and BP. Under warmer conditions, the combined increases in LMA/LC and LAI with Gst exceed the increase in BP (Figure 3e–g; Figure S7), resulting in a higher *η*_f_. Under drier conditions, although LAI declines more steeply than BP, the increase in LMA/LC with AI more than compensates for the reductions in LAI and BP (Figure 3f–h; Figure S7), also leading to an increase in *η*_f_. The model successfully reproduces the positive relationships between *η*_f_ and both Gst and AI but overestimates the sensitivity of *η*_f_ to aridity (Figure 3a,c). For deciduous plants, this bias may reflect an overestimation of the response of LMA/LC to aridity (Figure S7b). For evergreen plants, it may result from an underestimation of the decline in LAI_max_ with increasing aridity (Figure S7h).

Experimentally elevated CO_2_ (eCO_2_) enhances both leaf biomass and total plant biomass production (Figure 4a,b). In woody plants, the increase in total biomass generally exceeds that in leaf biomass, leading to a decline in the fraction of biomass allocated to leaves, whereas non–woody plants show the opposite pattern. Our model successfully captures these responses. At the global scale, eCO_2_ is predicted to reduce the fraction of biomass allocated to leaves in woody–dominated regions, while slightly increasing it in non–woody–dominated regions (Figure 4c–f). These different responses between woody– and non–woody–dominated regions are primarily driven by differences in water availability. Specifically, under humid conditions, eCO_2_ has an overall negative effect on *η*_f_ for both woody and non–woody plants. However, as dryness increases, the negative effect of elevated CO_2_ on *η*_f_ remains relatively stable in woody plants but becomes substantially more positive in non–woody plants (Figure S8). Because non–woody plants are predominantly distributed in drier environments, eCO_2_ exerts an overall positive effect on their *η*_f_. From the perspective of trait adaptation, the enhancement of *η*_f_ in non–woody–dominated regions is attributed to the stronger positive effect of elevated CO_2_ on LMA/LC relative to its negative effect on leaf area, whereas the opposite holds in woody–dominated regions (Figure 4g,h).

**FIGURE 4:**
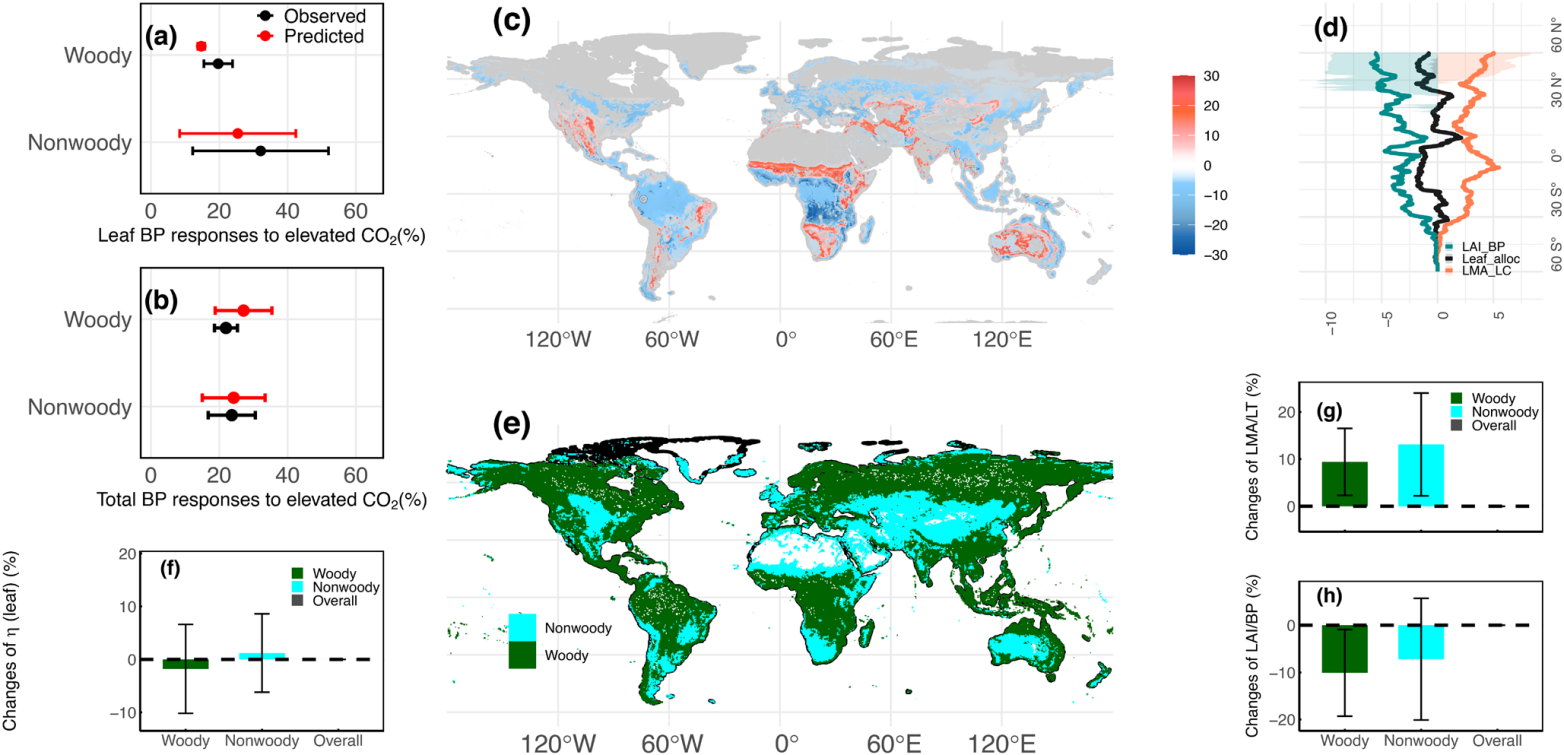
Predicted and observed effects of elevated CO_2_ on (a) leaf biomass production (BP) and (b) plant total BP. Symbols represent the mean percentage change from control (CO_2_ = 400 ppm). Bars show 95% bootstrapped confidence intervals (CIs). Predicted: Red. Observed: Black. Only statistically significant effects are shown. (c) The modelled change in fraction of biomass allocated to leaves under elevated CO_2_ (700 ppm) relative to 400 ppm. (d) Latitudinal variations of the changes in leaf carbon allocation, the ratio of LAI to BP, and the ratio of LMA to the leaf life cycle (LC: 365 days or leaf lifespan) under elevated CO_2_ (700 ppm) relative to 400 ppm. (e) The global distribution of nonwoody– and woody–dominated regions; (f–h) modelled effects of elevated CO_2_ for nonwoody– and woody–dominated regions.

### 2.4 Predicting Past Changes in Leaf Carbon Allocation

The model predicts that leaf C allocation has been declining over the past 120 years, with the rate of decrease accelerating (to −0.36% decade^−1^) after 2000 (Figure 5a). This acceleration is driven by a greater increase in BP relative to LAI (Figure 5e) under elevated CO_2_ conditions (Figure 5g; Figures S12–S14). Although plants tend to increase LMA/LC in response to elevated CO_2_ (Figure 5c,d; Figures S12–S14), this adjustment does not fully offset the reduction in leaf C allocation caused by the faster increase in BP compared with LAI. The enhanced increase in LMA/LC, together with the increase in BP and LAI since the 1950s (Figure 5c,e), suggests that leaf morphological adjustment has accelerated, particularly in woody–dominated regions (Figures S15 and S16). Simulated LAI_max_ and fAPAR time series (Cai et al. 2025; Zhou et al. 2025) show that the predicted leaf C allocation trends are consistent with changes in LAI (Figure S11), except for a slight overestimation of the decline in C allocation in woody–dominated regions (Figure S11b).

**FIGURE 5:**
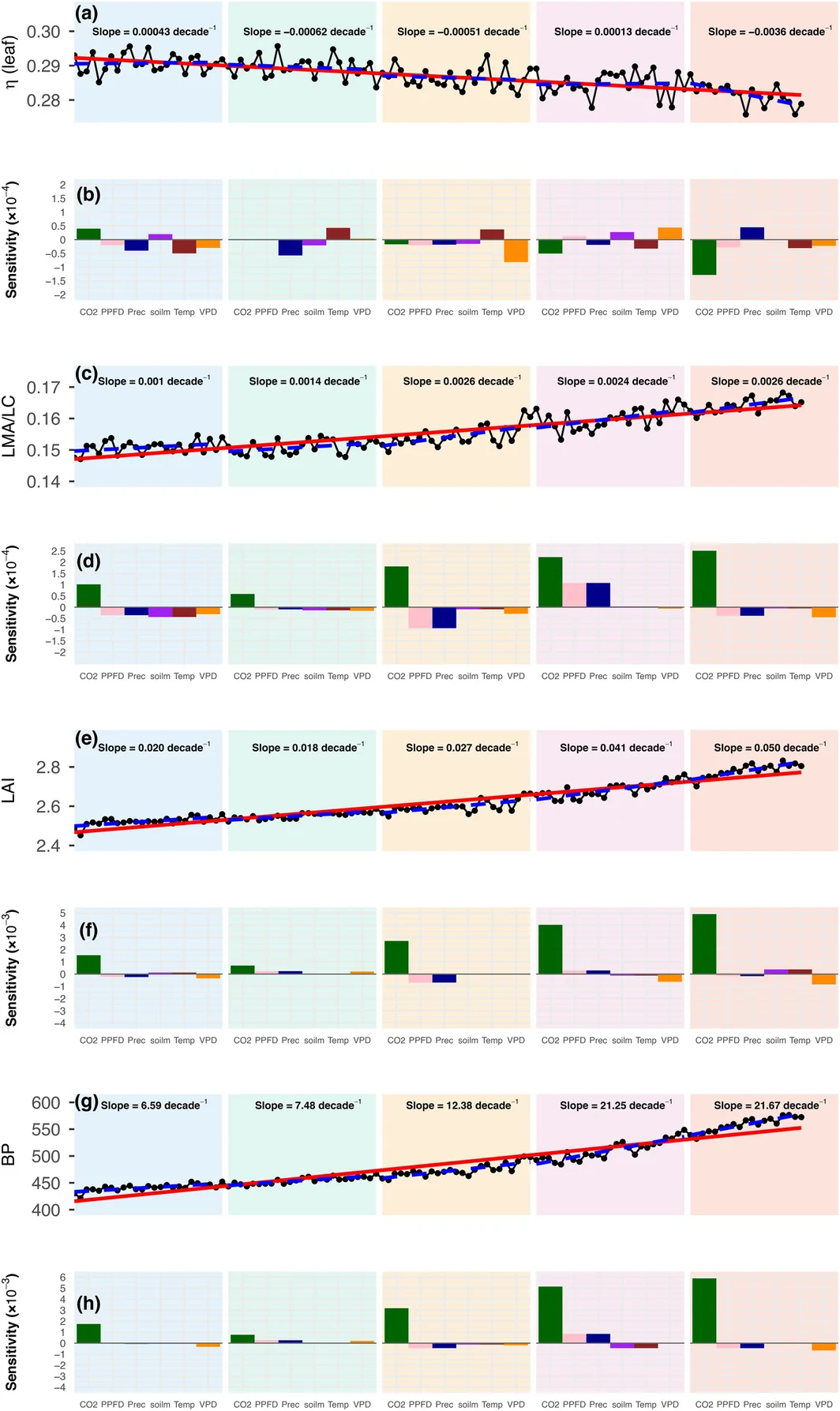
Global attribution of predicted trends (Theil–Sen slope coefficients) in (a) leaf carbon allocation (*η*(leaf)), (c) the ratio of leaf mass per area to leaf life cycle (LMA/LC), (e) seasonal maximum leaf area index (LAI) and (g) plant total biomass production (BP), to atmospheric carbon dioxide (CO_2_), photosynthetic photon flux density (PPFD), precipitation (Prec), soil moisture (soilm), temperature (Temp) and vapour pressure deficit (VPD). (b, d, f, h) show the contribution of each factor for each time range. Blue dashed lines are regression lines fitted for each time range; red solid lines are fitted across the whole range from 1901 to 2019.

## 3 Discussion

We have constructed an EEO–based framework to predict the fractional allocation of plant biomass production to leaves, using only environmental variables as inputs. The calculation is parsimonious, depending on three quantities—LAI, BP and LMA/LC—which we found to respond antagonistically to environmental change, contributing to the relative stability of leaf C allocation. These results support our hypothesis that coordinated adjustments in leaf economics, leaf area and total biomass production underlie this stability. They also suggest that changes in leaf C allocation cannot be evaluated solely from satellite–derived LAI (Li and Prentice 2024; Meng et al. 2023), but instead require accounting for environmentally driven variation in total biomass production as well as environmentally driven adjustments in leaf economic traits. Despite this overall consistency, the model's explanatory power remains moderate (*R* ^2^ = 0.33) and the observed–predicted relationship is relatively shallow (Figure 2a), indicating that substantial variation within woody and non–woody vegetation groups remains unexplained.

An important consideration is the timescale over which the predicted optimal state is achieved. The framework is formulated primarily at an annual timescale, with leaf C allocation calculated from annual biomass production and leaf biomass determined by LAI and LMA/LC. It therefore integrates processes operating across different timescales, from relatively rapid physiological responses to slower adjustments in leaf economic traits and canopy structure, which may occur over months to seasons. The predicted allocation should thus be interpreted as an integrated, longer–term allocation state that emerges as plant traits and canopy structure adjust to prevailing environmental conditions, while incorporating the cumulative effects of faster physiological responses. This framework is more appropriate for interpreting spatial patterns and long–term changes in allocation, rather than short–term responses to abrupt environmental perturbations.

One of the major environmental drivers of leaf C allocation responses is rising atmospheric CO_2_, which can enhance leaf photosynthesis and water–use efficiency via increasing light use efficiency (Ainsworth and Long 2005) and decreasing stomatal conductance (Leakey et al. 2009; Song et al. 2018). However, there is no consensus regarding the effects of elevated CO_2_ on C allocation. Some studies have indicated that elevated CO_2_ more strongly promotes belowground compared to aboveground C allocation (De Kauwe et al. 2014), likely because increased nutrient and/or water demands drive greater allocation to roots for their acquisition (Song et al. 2018). Conversely, Drake et al. (2019) found that experimental drought in young *Eucalyptus* trees increased C allocation aboveground to respiration and growth, while decreasing allocation belowground. This discrepancy may have arisen because enhanced water–use efficiency under elevated CO_2_ particularly benefits plants under drought (Fatichi et al. 2016; Song et al. 2018). Our results help reconcile these seemingly contrasting findings, indicating that the effect of elevated CO_2_ on leaf C allocation depends on water availability.

Moreover, woody and non–woody plants show different allocation responses to elevated CO_2_ under dry conditions, which may reflect their contrasting adaptation strategies (Breshears and Barnes 1999). The larger and deeper root system of woody plants is generally more effective than that of grasses in reaching deep soil–water stores, which can buffer the effects of drought (Chiatante et al. 1999; Morgan et al. 2007). It has also been found that the root C allocation rate of herbaceous plants is faster than that of woody plants under drought. This finding may, to some extent, explain why the leaf C allocation of herbaceous plants is more sensitive to drought than that of woody plants (Eziz et al. 2017; Xing et al. 2024).

Our framework broadly reproduces the difference in the drought response of foliar C allocation between herbaceous and woody plants, but the relatively large within–group variability indicates that the model does not fully capture the mechanisms underlying these contrasting allocation strategies. This unexplained variability may reflect both limitations in the available evaluation data and uncertainties in the model formulation. First, additional experimental data are needed to better characterise how herbaceous and woody plants adjust their leaf morphological and hydraulic traits and C allocation strategies under arid conditions. Second, the residual uncertainty also points to two potentially important sources of uncertainty in the model: the representation of biomass production and leaf economic traits.

One source of uncertainty in BP is biomass production efficiency (BPE), which determines the fraction of GPP converted into annual biomass production (BP). BPE values were derived here using an empirical function of latitude, annual mean temperature, annual total precipitation and stand age (Collalti et al. 2020) for woody species and imposed as a constant for herbaceous plants (He et al. 2020). BPE can also be affected by vegetation type, soil fertility and management (Campioli et al. 2015; He et al. 2020; Zhang et al. 2015) but due to different sources of input data, there has been controversy over its representation on a global scale (Collalti et al. 2020; Tang et al. 2019; Zhang et al. 2015). For example, one analysis based on site measurements indicated that BPE increases with annual mean temperature (MAT) (Collalti et al. 2020), while another based on eddy covariance data found that BPE decreases with MAT (Luo et al. 2025). Although the relatively limited range of BPE values may reduce its contribution to overall variation in leaf C allocation, uncertainties in its environmental dependence could contribute to the unexplained variation in Figure 2a. Moreover, given the limited understanding of how elevated CO_2_ influences BPE within current large–scale empirical frameworks, the direct effects of elevated CO_2_ on BPE were not explicitly incorporated in this study (Collalti et al. 2020; Luo et al. 2025).

Uncertainty also arises from the representation of leaf economic traits, particularly LMA/LC. In predicting LMA/LC, leaf–level carbon assimilation rates were scaled from canopy–level assimilation rates (Wang et al. 2023). This approach implicitly relies on a proportional relationship between leaf–specific and canopy–scale photosynthetic capacities (Sellers et al. 1996). Although this assumption provides a useful simplification for modelling purposes, this simple proportional relationship can be challenged by actual canopy structure and light attenuation processes (Dong, Prentice, et al. 2022; Sellers et al. 1996). For example, as canopy closure and LAI increase, light availability declines nonlinearly because of progressive self–shading within the canopy (Dong, Prentice, et al. 2022), reducing canopy–average photosynthetic capacity relative to that of individual leaves. Consequently, the relationship between leaf– and canopy–level photosynthesis may deviate from simple proportionality, introducing uncertainty into the prediction of LMA/LC. This uncertainty may be particularly relevant in dense canopies and could contribute to the weaker agreement between predicted and observed leaf C allocation within vegetation groups (Figure 2a).

Water limitation is another source of uncertainty in predicting LMA/LC. Its effects are incorporated empirically in the current framework. Although the model performs well, it is constrained by limited sample size and may not adequately capture the impacts of more frequent extreme weather under future climate change. Nevertheless, given that Wang et al. (2023) provides the first, and currently only, framework for predicting LMA/LC responses to environmental gradients, and that the model performs well against site–level observations, we consider it appropriate to use this framework to estimate leaf carbon allocation in the present study. In the future, a possible way to improve it is to explicitly consider how soil moisture regulates leaf water content and, in turn, LMA, as LMA has been shown to be closely linked to leaf water content (Wang, Huang, et al. 2022). Such limitations in representing the environmental responses of leaf economic traits may contribute to the unexplained variation in leaf C allocation evident in Figure 2a.

Beyond the processes directly represented in the model, nutrient availability may provide another source of variation in leaf C allocation. Nitrogen (N) is an essential nutrient, required for photosynthesis and many subsequent processes (Dong, Prentice, et al. 2022; Dong, Wright, et al. 2022; LeBauer and Treseder 2008; Stocker et al. 2025). However, there is no consensus on how N availability affects plant C allocation in response to changes in CO_2_ and climate. Experiments have shown that N fertilization predominantly increases leaf area, while N deposition strongly reduces LMA (Stocker et al. 2025). The link between these experimental findings and the behaviour of natural ecosystems remains unclear. Even though elevated CO_2_ can cause a shift towards belowground growth and biomass allocation, this shift in turn increases N uptake, constraining further belowground allocation (De Graaff et al. 2006; Jiang et al. 2020; Leakey et al. 2009; Schneider et al. 2004). The strength of this feedback is unknown (Stocker et al. 2025). Another study found that, as CO_2_ concentration and temperature increase, the optimal N content of plant leaves decreases. This finding can explain observed negative trends in leaf N and suggests that N limitation on BP under elevated CO_2_ may be alleviated—rather than increased, as some previous analyses proposed (Bassiouni et al. 2025; Dong, Wright, et al. 2022). Thus, although N availability likely influences foliar C allocation, its role in determining C allocation patterns across space, time and climatic regimes requires deeper investigation. In our model, for simplicity, the influence of N availability on leaf area and leaf economy was not considered, and the cost of leaf construction was estimated as a constant multiplier of LAI (Cai et al. 2025; Wang et al. 2023; Zhou et al. 2025).

Overall, our results demonstrate that leaf C allocation can be understood as an emergent property of the coordinated adjustment of leaf area, leaf economic traits and total biomass production under environmental constraints. The framework provides a parsimonious way to integrate these processes and predicts broad spatial and environmental patterns of leaf C allocation. At the same time, the relatively low explanatory power of the observed–predicted relationship highlights the importance of processes that are currently simplified or omitted, including variation in BPE, canopy structure, water limitation of leaf economic traits, and nutrient availability. Incorporating these processes as more observational constraints become available should improve the representation of leaf C allocation at the community level and its response to future climate and atmospheric CO_2_ change. Despite these limitations, our framework provides a theoretical basis for linking leaf economics and canopy structure with leaf C allocation at the community level and offers a tractable approach for incorporating environmentally responsive leaf C allocation into global vegetation models.

## 4 Materials and Methods

### 4.1 Leaf Carbon Allocation

The leaf C allocation prediction workflow has three steps (Figure S1): (1) Predict annual leaf biomass production (BP) by combining the leaf economics model (Wang et al. 2023) with the LAI model (Cai et al. 2025; Zhou et al. 2025). (2) Predict GPP by forcing a universal and extensively validated light use efficiency (LUE) model—the P model (Stocker et al. 2020; Wang et al. 2017) – with predicted fAPAR (the fraction of incident photosynthetically active radiation that is absorbed by leaves) timeseries (Zhou et al. 2025), then predict plant total BP from GPP via an empirical equation for BPE. (3) Calculate the annual fraction of BP allocated to leaves, as the ratio of leaf BP to plant total BP.

### 4.2 Leaf Economics

A theory based on EEO principles states that the proportional relationship between LMA and LL (the foundation of the worldwide leaf economics spectrum; Wright et al. 2004) can be quantified by maximizing average net C gain over the leaf life cycle (Wang et al. 2023). The theory yields different predictions for deciduous and evergreen plants (Equations 1 and 2) because deciduous leaves are replaced just once a year, with their lifespan tied to the length of the favourable season, whereas evergreen leaves are replaced continuously over a longer period:

(1) $${\mathrm{LL}}_{\mathrm{ev}}=\frac{\mathrm{LMA}}{\mathrm{nk}A_{0}}\sqrt{\frac{2uh_{T}m_{c}C_{c}}{f}}$$

(2) $${\mathrm{LL}}_{\mathrm{de}}=\frac{\mathrm{LMA}}{{\mathrm{nkA}}_{0}}\left(uh_{T}m_{c}\right)=365f$$

where subscripts ‘ev’ and ‘de’ denote evergreen and deciduous species, respectively; *n* = 2.5 g biomass g C^−1^; *A*_0_ is the potential GPP (g C m^−2^ ground area day^−1^) that would be achieved if fAPAR = 1, calculated from Equation 3.10; *k* is the light extinction coefficient (*k* = 0.5 m^−2^ leaf area m^−2^ ground area), which is used to scale canopy–level photosynthesis (*A*_0_, g C m^−2^ ground area day^−1^) to leaf–level photosynthesis (*kA*_0_, g C m^−2^ leaf area day^−1^) according to the Beer–Lambert law; *u* is a constant (*u* = 768) that relates the leaf ageing rate to LMA and carboxylation capacity (*V*_cmax_), estimated from a meta–analysis of data on species across temperate and tropical biomes (Wang et al. 2023; Xu et al. 2017); *h*_T_ is the Arrhenius function that relates *V*_cmax_ to temperature (*T*), equal to unity when *T* = 25°C; *m*_c_ is the ratio of the Rubisco−limited photosynthetic rate to *V*_cmax_, which is a function of the intercellular CO_2_ partial pressure; *C*_c_ is a dimensionless multiplier for the total plant–level C expenditure associated with leaf construction, maintenance and supply of water and nutrients, estimated as 1.57 gC gC^−1^ (Wang et al. 2023); and *f* is the growing–season duration, as a fraction of the year (> 0°C).

Both LMA and LL can be affected by water limitation. However, the increasing magnitude of the ratio LMA/LL with aridity was not fully integrated in the LMA theory (Wang et al. 2023). Here these effects were first estimated by multiple regression of log–transformed (observed) LMA/LL against log–transformed LMA/LL as predicted by Equations 1 or 2, and the log−transformed climatological Aridity Index (AI):

(3) $$\ln{\left(\frac{\mathrm{LMA}}{\mathrm{LL}}\right)}_{\text{water}}=\ln{\left(\frac{\mathrm{LMA}}{\mathrm{LL}}\right)}_{\text{nonwater}}+a\times \ln\mathrm{AI}+b$$

where the subscripts ‘water’ and ‘nonwater’ indicate whether or not aridity effects are considered. AI here is the ratio of mean annual potential evapotranspiration to mean annual precipitation (Good et al. 2017), calculated using the Simple Process−led Algorithms for Simulating Habitats (SPLASH) model (version 1) (Davis et al. 2017). The coefficient *a* was estimated as 1.08 for deciduous (*a*_de_) and 0.60 for evergreen plants (*a*_ev_), and b was estimated as 0.88 for deciduous (*b*_de_) and −0.41 for evergreen plants (*b*_ev_).

### 4.3 Seasonal Maximum LAI and LAI Dynamics

Seasonal maximum leaf area index (LAI_max_) governs the annual biomass allocated to foliage (Equation 13). LAI_max_ can be related to the seasonal peak fraction of absorbed photosynthetically active radiation (fAPAR_max_) (Cai et al. 2025) via the Beer's law approximation (Equation 5). fAPAR_max_ was predicted based on eco–evolutionary optimality principles, whereby canopy development is constrained either by energy availability (in which case fAPAR_max_ is determined via a C profit–maximization principle) or by a hydrological limit imposed by the transpiration demands of photosynthetic gas exchange (Cai et al. 2025):

(4) $${\text{fAPAR}}_{\max}=\min\;\left\{1-z/\left(kA_{0}\right),\left[c_{a}\;\left(1-\chi \right)/1.6\;D\right]\;\left[f_{0}\;P/A_{0}\right]\right\}$$

(5) $${\mathrm{LAI}}_{\max}=-\left(1/k\right)\;\ln\;\left(1-{\text{fAPAR}}_{\max}\right)$$

In Equation 4, *z* represents the costs of constructing and maintaining leaves and supplying them with water and nutrients and has a global fitted value of 12.227 mol m^−2^ a^−1^ (Cai et al. 2025); *k* is the light extinction coefficient (set at 0.5); *c*_a_ is the ambient CO_2_ partial pressure (Pa); *χ* is the ratio of leaf–internal CO_2_ partial pressure to *c*_a_; *D* is the mean vapour pressure deficit (VPD) during the growing season (Pa); *f*_0_ is the ratio of transpiration to annual total precipitation; and *P* is total annual precipitation (mol m^−2^ year^−1^). More details about the calculation of *f*_0_ are provided in Table S1.

An extended version of the model of Cai et al. (2025) was used to simulate seasonal LAI dynamics (Zhou et al. 2025). This model is built on an assumed universal relationship between ‘steady–state’ GPP and LAI. This steady state represents the mutually consistent LAI and GPP expected under constant conditions (Equation 6). Inherent time lags in the phenological response to weather fluctuations impose a delay between the steady–state LAI and the real–time dynamic LAI. The exponential weighted moving average method (Equation 7) was used to incorporate this lag effect (Zhou et al. 2025). This prognostic and globally applicable model, which relies solely on climate and CO_2_ data inputs, has been shown to capture satellite–derived LAI dynamics across biomes at both site and global levels (Zhou et al. 2025):

(6) $${\mathrm{LAI}}_{s}=\min\left\{\mu +\left(\frac{1}{k}\right)W_{0}\;\left[-\mathrm{k\mu}\;\exp\;\left(-\mathrm{k\mu}\right)\right],{\mathrm{LAI}}_{\max}\right\}$$

(7) $${\mathrm{LAI}}_{\mathrm{sim}}\left[t\right]=\alpha \times {\mathrm{LAI}}_{s}\left[t\right]+\left(1-\alpha \right)\times {\mathrm{LAI}}_{\mathrm{sim}}\left[t-1\right]$$

where LAI_s_ denotes the steady–state LAI; *W*_0_ is the principal branch of the Lambert W function; *μ* = *mA*_0_, with *m* estimated according to Table S1; and LAI_sim_ is a weighted average of the steady–state LAI value corresponding to the steady–state GPP of the day (LAI_s_) and its acclimated value from the previous day, with weightings of *α* and (1 − *α*) respectively. *α* is set to 0.067, corresponding to approximately 15 days of memory.

### 4.4 Plant Total Biomass Production

Biomass production efficiency (BPE) quantifies how efficiently assimilated C is allocated to BP. The BPE of woody plants was predicted from an empirical equation of absolute latitude, precipitation, temperature and stand age (Collalti et al. 2020) (Equation 8) while the BPE of grassland was set to 0.45 (He et al. 2020):

(8) $$\mathrm{BPE}=0.19+0.006\;\mathrm{MAT}-0.00038\;\mathrm{age}+0.000068\;\mathrm{TAP}+0.0039\;\left|\mathrm{lat}\right|$$

where MAT is mean annual temperature (°C); age is forest stand age (years); TAP is total annual precipitation (mm year^−1^); and |lat| is absolute latitude (°). GPP was multiplied by BPE to yield plant total BP:

(9) Plant biomass production=GPP×BPE

GPP (A) was estimated as the product of fAPAR, Light Use Efficiency (LUE) and photosynthetic photon flux density (PPFD):

(10) A=fAPAR×LUE×PPFD

In Equation 10, LUE is given by:

(11) $$\mathrm{LUE}=\varphi_{0}\;M\;\sqrt{1-{\left(\frac{c^{*}}{M}\right)}^{\frac{2}{3}}}$$

(12) $$M=\frac{c_{a}-\Gamma^{*}}{\left\{c_{a}+2\Gamma^{*}+3\Gamma^{*}\sqrt{\left[1.6\eta^{*}D\beta^{-1}{\left(K+\Gamma^{*}\right)}^{-1}\right]}\;\right\}}$$

where *φ*_0_ is the intrinsic quantum yield of photosynthesis (mol CO_2_ mol^−1^ photon), *M* represents the impact of leaf–internal CO_2_ on C assimilation. *c*_a_ is the ambient CO_2_ partial pressure (Pa), Γ* is the CO_2_ compensation point in the absence of mitochondrial respiration (Pa), *K* is the effective Michaelis–Menten coefficient of Rubisco (Pa), η* is the viscosity of water relative to its value at 25°C (dimensionless), *β* is the ratio of cost factors for carboxylation and transpiration at 25°C, estimated as 146, and c* is a cost factor for electron transport capacity, estimated as 0.41 (Stocker et al. 2020). *φ*_0_, Γ*, *K* and η* depend on temperature and Γ* and *K* on atmospheric pressure, as described by Stocker et al. (2020).

### 4.5 Leaf Carbon Allocation

The flux of biomass to leaves was calculated as the total leaf biomass divided by the leaf life cycle (LC, days) (Equation 13). Here, LC refers to the period from leaf formation to leaf replacement. Thus, LC is equal to LL for evergreen species, whereas it is set to 365 days for deciduous species to represent the annual turnover of leaves. Leaf biomass was taken to be the product of LMA and LAI. Seasonal maximum LAI (LAI_max_) was employed in this calculation since it determines annual foliar biomass allocation:

(13) $$\text{Leaf biomass growth}={\mathrm{LAI}}_{\max}\times {\left(\frac{\mathrm{LMA}\;}{\mathrm{LC}}\right)}_{\text{water}}$$

By combining Equations 9 and 13, we obtain a general expression for leaf C allocation in evergreen plants (Equation 14) and deciduous plants (Equation 15):

(14) $$\eta {\left(f\right)}_{\mathrm{ev}}=\frac{{\mathrm{LAI}}_{\max}}{\mathrm{GPP}\times \mathrm{BPE}}\times {\left(\frac{\mathrm{LMA}}{\mathrm{LL}}\right)}_{\text{water}}$$

(15) $$\eta {\left(f\right)}_{\mathrm{de}}=\frac{{\mathrm{LAI}}_{\max}}{\mathrm{GPP}\times \mathrm{BPE}}\times {\left(\frac{\mathrm{LMA}}{365}\right)}_{\text{water}}$$

Substituting Equations (1), (2), (3) into Equations 14 and 15, we obtain general equations for the fraction of biomass allocated to leaves in evergreen (Equation 16) and deciduous (Equation 17) plants:

(16) $$\eta {\left(f\right)}_{\mathrm{ev}}=\frac{{\mathrm{LAI}}_{\max}}{\mathrm{GPP}\times \mathrm{BPE}}\times \left({\mathrm{nkA}}_{0}\sqrt{\frac{f}{2uh_{T}m_{c}C_{c}}}\;\right)\times \exp\left(a_{\mathrm{ev}}\ln\mathrm{AI}+b_{\mathrm{ev}}\right)$$

(17) $$\eta {\left(f\right)}_{\mathrm{de}}=\frac{{\mathrm{LAI}}_{\max}}{\mathrm{GPP}\times \mathrm{BPE}}\times \left(\frac{{\mathrm{nkA}}_{0}f}{uh_{T}m_{c}}\right)\times \exp\left(a_{\mathrm{de}}\ln\mathrm{AI}+b_{\mathrm{de}}\right)$$

As *A*_0_ is the GPP that would be achieved if fAPAR = 1, actual GPP can be represented as GPP = *A*_0_ × fAPAR, yielding:

(18) $$\eta {\left(f\right)}_{\mathrm{ev}}=\frac{{\mathrm{LAI}}_{\max}}{\text{fAPAR}\times A_{0}\times \mathrm{BPE}}\times \left({\mathrm{nkA}}_{0}\sqrt{\frac{f}{2uh_{T}m_{c}C_{c}}}\;\right)\times \exp\left(a_{\mathrm{ev}}\ln\mathrm{AI}+b_{\mathrm{ev}}\right)$$

(19) $$\eta {\left(f\right)}_{\mathrm{de}}=\frac{{\mathrm{LAI}}_{\max}}{\text{fAPAR}\times A_{0}\times \mathrm{BPE}}\times \left(\frac{\mathrm{nk}A_{0}f}{uh_{T}m_{c}}\right)\times \exp\left(a_{\mathrm{de}}\ln\mathrm{AI}+b_{\mathrm{de}}\right)$$

and after simplification, eliminating *A*_0_:

(20) $$\eta {\left(f\right)}_{\mathrm{ev}}=\frac{{\mathrm{LAI}}_{\max}}{\text{fAPAR}\times \mathrm{BPE}}\times \mathrm{nk}\sqrt{\frac{f}{2uh_{T}m_{c}C_{c}}}\times \exp\left(a_{\mathrm{ev}}\ln\mathrm{AI}+b_{\mathrm{ev}}\right)$$

(21) $$\eta {\left(f\right)}_{\mathrm{de}}=\frac{{\mathrm{LAI}}_{\max}}{\text{fAPAR}\times \mathrm{BPE}}\times \left(\frac{\mathrm{nkf}}{uh_{T}m_{c}}\right)\times \exp\left(a_{\mathrm{de}}\ln\mathrm{AI}+b_{\mathrm{de}}\right)$$

### 4.6 Study Sites and Sampling

Five site measurement datasets were used in our study (Lu et al. 2025; Peng et al. 2023; Van Sundert et al. 2023; Wang, Harrison, et al. 2022; Wright et al. 2004). We used two nonoverlapping datasets, the China Plant Trait Database version 2 (CPTD) (Wang, Harrison, et al. 2022) and the Global Plant Trait Network (Glopnet) dataset (Wright et al. 2004) to investigate the variation of deciduous species' LMA with environment (Figure 2e–f; Figures S2a, S3a,c, and S7a,b). Predictions of the ratio of evergreen species' LMA to their leaf lifespan (Figure 2g,h; Figures S2b, S3b,d, and S7c,d) were evaluated based on Glopnet, which uniquely contains paired LMA and LL measurements.

We combined two independent sites from C allocation datasets (Lu et al. 2025; Peng et al. 2023) to evaluate the predicted annual fraction of biomass allocated to leaves (Figure 2; Figures S3a–d and S5). The resulting comprehensive dataset includes 503 canopy measurements, comprising 412 sites dominated by woody plants and 81 sites dominated by non–woody plants (Figure S18). Among the woody sites, 326 are dominated by evergreens, while 86 support deciduous woody vegetation.

Predicted effects of elevated CO_2_ on leaf C allocation were evaluated using the Manipulation Experiments Synthesis Initiative (MESI) dataset (Van Sundert et al. 2023) (Figure 4; Figure S10). The subset we used includes 145 records, 41 for leaf BP and 104 for plant total BP.

### 4.7 Gridded Climate and Satellite Data

We downloaded six–hourly temperature (°C), precipitation (mm), surface downwelling shortwave radiation (W m^−2^), atmospheric pressure (Pa) and specific humidity (g kg^−1^) from the CRU–JRA dataset version 2.5 (Harris et al. 2020) for 1901–2019 at a grid resolution of 0.5°. These data were converted to daily timescales, as inputs to the P model, by averaging (Stocker et al. 2020). VPD (Pa) was calculated from daily mean temperature and specific humidity. Solar radiation was converted to incident PPFD assuming a flux: energy ratio of 4.6 μmol J^−1^ and a photosynthetically active fraction of 0.5. We downloaded the 1901–2019 global monthly mean CO_2_ concentrations (μmol mol^−1^) from NOAA Global Monitoring Laboratory for analysis (NOAA/GML; https://gml.noaa.gov/ccgg/trends/; last access October 2024). For site–level analysis, we extracted and intercepted the climate data from the CRU–JRA dataset version 2.5 (Harris et al. 2020) during the period between 1982 and 2010 in order to match the times when the leaf data were recorded (Lu et al. 2025; Peng et al. 2023; Van Sundert et al. 2023; Wang, Harrison, et al. 2022; Wright et al. 2004). For global analysis, the whole time period (1901–2019) was used.

Canopy–level potential GPP (*A*_0_), the Arrhenius function for *V*_cmax_ (*h*_T_) and the ratio of the Rubisco–limited photosynthetic rate to *V*_cmax_ (*m*_c_) were calculated from the P model (Stocker et al. 2020) with daily mean temperature, PPFD, VPD, atmospheric pressure and CO_2_ concentration as inputs. The ratio of leaf–internal CO_2_ partial pressure to ambient CO_2_ concentration (*χ*) was calculated following Prentice et al.'s (2014) method. As the standard P model requires separate calculations of GPP for C3 and C4 photosynthetic pathways, a dynamic C4 vegetation fraction was simulated based on a C3/C4 competition model (https://pyrealm.readthedocs.io/en/latest/) embedded in the P model. Soil moisture stress effects on simulated A₀ were incorporated using the empirical penalty factor (Stocker et al. 2020), with the annual time course of soil moisture calculated using the Simple Process–led Algorithms for Simulating Habitats (SPLASH) model (version 1) (Davis et al. 2017). Annual mean potential evapotranspiration was also obtained from the SPLASH model to calculate the Aridity Index (AI).

The calculation of BPE for woody species requires forest age as input (Equation 8). As only a few sites in the C allocation dataset (Lu et al. 2025; Peng et al. 2023) have measured tree age data, we extracted forest age data from a global forest age dataset (GFADv1.0) at a grid resolution of 0.5° (Poulter et al. 2018) as the mean age across four plant functional types (PFTs) and 15 age classes (from 0–10 to 140–150 years), weighted by their respective fractional area coverage within each grid cell.

Seasonal maximum LAI (Equations 4 and 5) and fAPAR dynamics (Equations 6 and 7) were predicted from the seasonal maximum fAPAR model (Cai et al. 2025) and a global prognostic LAI model (Zhou et al. 2025). In order to exclude any potential impacts of LAI/fAPAR prediction on the performance of the leaf C allocation framework, we also extracted annual peak LAI and daily fAPAR data from the MODIS MOD15A2H Leaf Area Index/FPAR product from 2001 to 2019 (Li et al. 2023) as supplementary datasets.

As evergreen and deciduous plants have to be considered in different ways (Equations 20 and 21), the simulation of leaf C allocation on a global level needs to distinguish deciduous–dominated and evergreen–dominated areas. Regarding woody species, two kinds of global land cover map were used to distinguish deciduous– and evergreen–dominated areas. The first comes from the ESA CCI–LC database (https://www.esa‐landcover‐cci.org/?q=node/164) at a resolution of 300 m (Defourny et al. 2023). In this land cover map, areas dominated by mixed forest were treated as half evergreen species and half deciduous. Another comes from a semi–empirical dataset (Li and Prentice 2024) that estimates the proportion of deciduous and evergreen plants within each land cover class based on surface observations (Sabatini et al. 2021), then extrapolates these proportions globally using the ESA CCI–LC global land cover map. Unlike the ESA CCI–LC dataset (Defourny et al. 2023), this dataset provides percentages of deciduous and evergreen vegetation, rather than a binary classification. Perennial grasslands (maintaining green foliage year–round) and seasonally dormant grasslands (undergoing dormancy or shedding) were classified according to the following steps. First, we determined the growing season length (GSL) for each grassland site using smoothed LAI data. The growing season was identified with the bigleaf package (Knauer et al. 2018), which defines vegetation as active when its LAI exceeds 5% of the annual peak (95th percentile). Then areas with the GSL larger than 0.95 were classified as perennial grassland, while those below were considered seasonally dormant (Figure S17). Deciduous woody plants and seasonally dormant grasslands were uniformly named as seasonally senescent plants, and evergreen woody plants and perennial grasslands were uniformly named as persistent perennial plants.

### 4.8 Data Analysis

#### 4.8.1 Evaluation of Leaf Economics Predictions

Ordinary least squares multiple linear regression (OLS) in the lme4 package (R Core Team) was used to investigate the expected increase in LMA and LC with aridity, as an additional constraint on LMA framework (Wang et al. 2023) (Figure 1). A mixed–effects model was also used to study the aridity constraint on LMA and LC, as a supplementary analysis to exclude the random effects of site (Figure S2). After adding the aridity constraints to the original LMA framework (Wang et al. 2023), the ability of this model to predict leaf economics was effectively evaluated at site level (Figure 1; Figure S3).

#### 4.8.2 Evaluation of Leaf Carbon Allocation Predictions

We first assessed the predictive accuracy of the proposed leaf C allocation model at the site level (Figure 2a) and subsequently examined its capacity to replicate observed trends in leaf C allocation dynamics across environmental gradients (Figures 3a–d and 4a,b).

To identify key factors that can potentially affect leaf C allocation, we first selected five variables: four critical environmental factors influencing leaf economics—growing season mean temperature (Gst), total growing season photosynthetic photon flux density (*I*_abs_), growing season length (*f*) and the aridity index (AI, defined as the ratio of mean annual potential evapotranspiration to precipitation) (Wang et al. 2023), alongside stand age, a physiological variable linked to plant developmental stages (Dolezal et al. 2021; Song et al. 2018). The selection of factors (Table S2) was performed by forward stepwise regression, adding the variable producing the largest increase in *R* ^2^ at each step. All retained predictors were required to have statistically significant regression coefficients (*p* < 0.05, evaluated via *t*‐tests). The selection process terminated when no additional variables met this significance criterion. All factors (*I*_abs_, *f*, AI, age), except for Gst, were log–transformed. Two factors (Gst and AI) were selected (Figure 3).

Given the well–documented negative effects of elevated CO_2_ (eCO_2_) on leaf C allocation (De Kauwe et al. 2014; Duanmu et al. 2025) and its important role in driving leaf area (Cai et al. 2025), we also tested the ability of the leaf C allocation framework presented here in simulating elevated CO_2_ effects (Figure 4). As MESI (Van Sundert et al. 2023) provides the impact of elevated CO_2_ on leaf BP and total plant biomass, we focused on testing the model's performance in capturing CO_2_–driven changes in these quantities (Figure 4a,b). We evaluated these effects through a meta–analysis conducted in MetaWin 2.1.3. The ln response ratio (RR), calculated as RR = ln(Xt/Xc), was employed to quantify the changes in leaf and plant total biomass under CO_2_–driven conditions (Agathokleous et al. 2021; Zhou et al. 2022), where Xt and Xc represent the means of leaf BP and plant total BP under elevated CO_2_ and control treatments (CO_2_ concentration = 400 ppm), respectively. The weighted response ratio and bias–corrected 95% bootstrap–confidence interval (CI) were calculated using a random–effects model which accounts for both within–study sampling error and between–study heterogeneity in effect sizes (Xie et al. 2014). The predicted responses of leaf BP and plant BP to elevated CO_2_ were also calculated following the same equation for better comparison (Figure 4a,b). After verifying the capacity of our framework to simulate CO_2_–enrichment impacts on foliar carbon partitioning, we extended the analysis to examine global–scale implications of elevated atmospheric CO_2_ (fixed at 700 ppm concentration) on C allocation (Figure 4c). The division of woody–dominated and nonwoody–dominated areas (Figure 4e–h) was based on the ESA CCI–LC global land cover map (Defourny et al. 2023).

#### 4.8.3 Adaptation Rules of Leaf Carbon Allocation

We also tried to explain the governing mechanisms of the adaptation of leaf C allocation to environmental gradients (Figures 3e–h and 4d) and the changes over time (Figure 5) from three complementary dimensions: (1) leaf economics, quantified by the leaf mass per area–to–leaf life cycle ratio (LMA/LC), which reflects investments in thicker/denser leaves with prolonged longevity; (2) leaf area dynamics (LAI), indicating shifts in canopy–level leaf area allocation; and (3) plants' total biomass production (BP), capturing whole–plant changes in biomass production. The ability of our model to capture the observed trends in these three complementary dimensions was also evaluated (Figure S7).

#### 4.8.4 Trends of Leaf Carbon Allocation and Its Drivers

After verifying the ability of the proposed leaf C allocation framework to capture observed trends in leaf C allocation across environmental gradients (Figures 3a–d and 4a,b), we investigated the changes in leaf C allocation over a 120–year period (1901–2019) and its driving forces (Figure 5; Figures S15 and S16).

Our leaf C allocation framework uses simulated LAI_max_ and fAPAR time series (Cai et al. 2025; Zhou et al. 2025) as inputs (Equation 13). To address potential uncertainties in the trends of simulated LAI and fAPAR, we firstly cross‐verified the predicted trends during 2001 and 2019 using the MODIS LAI/fAPAR observation dataset (Li et al. 2023) as inputs (Figure S11). The driving forces of leaf C allocation trends during the study period (1901–2019) were identified following these steps: (1) The study period (1901–2019) was divided into five intervals (1901–1925, 1926–1950, 1951–1975, 1976–2000, 2001–2019) to evaluate shifts in primary drivers across distinct periods. (2) Main input environmental variables of our leaf C allocation framework (Figure S1)—temperature, solar radiation, precipitation, soil moisture, VPD and CO_2_—were sequentially fixed at the value of initial year during each interval while allowing other variables to vary. (3) Trends derived from these constrained simulations were compared to the fully forced scenario (all variables varying) to provide a measure of the effect of single drivers (Figure 5).

## Author Contributions

**Boya Zhou:** writing – original draft, validation, methodology, visualization, writing – review and editing, investigation, formal analysis, conceptualization, software, project administration, data curation. **I. Colin Prentice:** conceptualization, funding acquisition, writing – review and editing, supervision, resources, methodology.

## Funding

This work was supported by the Schmidt Sciences LLC (G‐21‐61881).

## Conflicts of Interest

The authors declare no conflicts of interest.

## Supporting information


**Table S1:** Definitions and expressions/values of parameters used in our leaf carbon allocation framework.
**Table S2:** The selection of key factors that affect leaf carbon allocation using forward stepwise regression.
**Table S3:** Summarized statistics from the regression of observed and predicted leaf carbon allocation against bioclimatic variables for woody plants and nonwoody plants separately.
**Figure S1:** Workflow of the Leaf Carbon Allocation framework.
**Figure S2:** Regression of the ratio of LMA to 365 days residuals for deciduous plants and the ratio of Leaf mass per area (LMA) to Leaf lifespan (LL) residuals for evergreen plants against log–transformed Aridity Index (AI, the ratio of mean annual potential evapotranspiration to mean annual precipitation) at the individual plant level.
**Figure S3:** Linear regressions between observed log–transformed ratio of leaf mass per area (LMA) to 365 days and predicted log–transformed LMA/365 for deciduous plants, and observed log–transformed ratio of LMA to leaf lifespan (LL) and predicted log–transformed LMA/LL for evergreen plants after considering the aridity constraint on LMA and LL.
**Figure S4:** Linear regressions of (a) observed and predicted ratios of leaf mass per area to leaf lifespan and (b) their bias, against seasonal maximum leaf area index.
**Figure S5:** Predicted (red) and observed (blue) distributions of the fraction of biomass production allocated to leaves *η*(leaf), for (a) seasonally senescent plants (deciduous woody and seasonally dormant non–woody species) and (b) persistent perennial plants (evergreen woody and persistent perennial non–woody species).
**Figure S6:** The spatial distribution of residuals between predicted and measured fraction of biomass allocated to leaves.
**Figure S7:** (a–d) Partial residual plots for the log–transformed ratio of Leaf Mass per Area (LMA) to Leaf Cycle (LC) against environmental variables. (e–h) Partial residual plots for the log–transformed Leaf Area Index (LAI) against environmental variables. (i–l) Partial residual plots for the log–transformed biomass production (BP) against environmental variables.
**Figure S8:** Changes in the response of leaf carbon allocation to elevated CO_2_ along the aridity index (AI) for (a) woody–dominated regions and (b) nonwoody–dominated regions.
**Figure S9:** Global maps of the predicted fraction of biomass allocated to leaves in 2010 based on different input LAI/fAPAR datasets and different land cover datasets.
**Figure S10:** Geographical distribution of the 145 elevated CO_2_ experiments included in the analysis.
**Figure S11:** Trends of predicted global mean leaf carbon allocation based on MODIS LAI/fAPAR (Li et al. 2023) as input, and on simulated LAI/fAPAR from a prognostic LAI model (Zhou et al. 2025) as input.
**Figure S12:** Trends of (a) annual mean temperature (K), (b) Carbon dioxide concentration (CO_2_, ppm), (c) annual total precipitation (mm year^−1^), (d) annual mean vapour pressure deficit (VPD, pa) and (e) soil moisture from 1901 to 2019 on a global level.
**Figure S13:** Trends of (a) annual mean temperature (K), (b) annual total precipitation (mm year^−1^), (c) annual mean vapour pressure deficit (VPD, pa) and (d) soil moisture from 1901 to 2019 for woody–dominated regions.
**Figure S14:** Trends of (a) annual mean temperature (K), (b) annual total precipitation (mm year^−1^), (c) annual mean vapour pressure deficit (VPD, pa) and (d) soil moisture from 1901 to 2019 for nonwoody–dominated regions.
**Figure S15:** Attribution of trends in (a) leaf carbon allocation (*η*(leaf)), (c) ratio of Leaf Mass per Area to Leaf Life Cycle (LMA/LC), (e) ratio of Leaf Area Index to Plant total Biomass production (LAI/BP) to various factors in woody–dominated regions.
**Figure S16:** Attribution of trends in (a) leaf carbon allocation (*η*(leaf)), (c) ratio of Leaf Mass per Area to Leaf Life Cycle (LMA/LC), (e) ratio of Leaf Area Index to Plant total Biomass production (LAI/BP) to various factors in nonwoody–dominated regions.
**Figure S17:** The global distribution of perennial grassland and seasonal dormant grassland.
**Figure S18:** Geographical distribution of the 483 sites included in this analysis.

## Data Availability

The data and code supporting the findings of this study are available on Zenodo at https://doi.org/10.5281/zenodo.21538563. The China Plant Trait Database (CPTD) version 2 and the Global Plant Trait Network (GLOPNET) trait dataset are publicly available at https://doi.org/10.1038/s41597‐022‐01884‐4 and https://doi.org/10.6084/m9.figshare.21800153, respectively. The leaf carbon allocation datasets are available from Peng et al. (2023) (https://doi.org/10.5281/zenodo.8182205) and Lu et al. (2025) (https://doi.org/10.1111/jbi.15094). MODIS leaf area index (LAI) and fraction of absorbed photosynthetically active radiation (fAPAR) data were obtained from the MODIS MOD15A2H product (Li et al. 2023). Climate data were obtained from the CRU‐JRA version 2.5 dataset, available at https://catalogue.ceda.ac.uk/uuid/aed8e269513f446fb1b5d2512bb387ad.
