# Supplementary material for: Optimal Biomass Allocation to Leaves Across Global Terrestrial Ecosystems
Source: Glob Chang Biol. 2026 Oct 28;32(11):e71166. doi: 10.1111/gcb.71166 (PMC13717054; doi:10.1111/gcb.71166)
Supplement: Supplementary file 1 [file GCB-32-e71166-s001.docx]

**Optimal biomass allocation to leaves across global terrestrial ecosystems**

Boya Zhou^1^, and I. Colin Prentice^1^,^2^

^1^Georgina Mace Centre for the Living Planet, Department of Life Sciences, Imperial College London, Silwood Park Campus, Buckhurst Road, Ascot SL5 7PY, UK; ^2^Department of Earth System Science, Ministry of Education Key Laboratory for Earth System Modelling, Institute for Global Change Studies, Tsinghua University, Beijing, China.

**Tables**

**Supplementary Table 1.** Definitions and expressions/values of parameters used in our leaf carbon allocation framework.

**Supplementary Table 2.** The selection of key factors that affect leaf carbon allocation using forward stepwise regression.

**Supplementary Table 3.** Summarized statistics from the regression of observed and predicted leaf carbon allocation against bioclimatic variables for woody plants and nonwoody plants separately.

**Figures**

**Supplementary Fig. 1.** Workflow of the Leaf Carbon Allocation framework.

**Supplementary Fig. 2.** Regression of the ratio of LMA to 365 days residuals for deciduous plants and the ratio of Leaf mass per area (LMA) to Leaf lifespan (LL) residuals for evergreen plants against log–transformed Aridity Index (AI, the ratio of mean annual potential evapotranspiration to mean annual precipitation) at the individual plant level.

**Supplementary Fig. 3.** Linear regressions between observed log–transformed ratio of leaf mass per area (LMA) to 365 days and predicted log–transformed LMA/365 for deciduous plants, and observed log–transformed ratio of LMA to leaf lifespan (LL) and predicted log–transformed LMA/LL for evergreen plants after considering the aridity constraint on LMA and LL.

**Supplementary Fig. 4.** Linear regressions of (a) observed and predicted ratios of leaf mass per area to leaf lifespan, and (b) their bias, against seasonal maximum leaf area index.

**Supplementary Fig. 5**. Predicted (red) and observed (blue) distributions of the fraction of biomass production allocated to leaves *η*(leaf), for **(a)** seasonally senescent plants (deciduous woody and seasonally dormant non–woody species) and **(b)** persistent perennial plants (evergreen woody and persistent perennial non–woody species).

**Supplementary Fig. 6.** The spatial distribution of residuals between predicted and measured fraction of biomass allocated to leaves.

**Supplementary Fig. 7. (a)–(d)** Partial residual plots for the log–transformed ratio of Leaf Mass per Area (LMA) to Leaf Cycle (LC) against environmental variables. **(e)–(h)** Partial residual plots for the log–transformed Leaf Area Index (LAI) against environmental variables. **(i)–(l)** Partial residual plots for the log–transformed biomass production (BP) against environmental variables.

**Supplementary Fig. 8.** Changes in the response of leaf carbon allocation to elevated CO₂ along the aridity index (AI) for **(a)** woody–dominated regions and **(b)** nonwoody–dominated regions.

**Supplementary Fig. 9.** Global maps of the predicted fraction of biomass allocated to leaves in 2010 based on different input LAI/fAPAR datasets and different land cover datasets.

**Supplementary Fig. 10.** Geographical distribution of the 145 elevated CO_2_ experiments included in the analysis.

**Supplementary Fig. 11.** Trends of predicted global mean leaf carbon allocation based on MODIS LAI/fAPAR (Li et al., 2023) as input, and on simulated LAI/fAPAR from a prognostic LAI model (Zhou et al., 2025) as input.

**Supplementary Fig. 12.** Trends of **(a)** annual mean temperature (K), **(b)** Carbon dioxide concentration (CO_2_, ppm), **(c)** annual total precipitation (mm year^–1^), **(d)** annual mean vapour pressure deficit (VPD, pa), and **(e)** soil moisture from 1901 to 2019 on a global level.

**Supplementary Fig. 13.** Trends of **(a)** annual mean temperature (K), **(b)** annual total precipitation (mm year^–1^), **(c)** annual mean vapour pressure deficit (VPD, pa), and **(d)** soil moisture from 1901 to 2019 for woody–dominated regions.

**Supplementary Fig. 14.** Trends of **(a)** annual mean temperature (K), **(b)** annual total precipitation (mm year^–1^), **(c)** annual mean vapour pressure deficit (VPD, pa), and **(d)** soil moisture from 1901 to 2019 for nonwoody–dominated regions.

**Supplementary Fig. 15.** Attribution of trends in (a) leaf carbon allocation (*η*(leaf)), (c) ratio of Leaf Mass per Area to Leaf Life Cycle (LMA/LC), (e) ratio of Leaf Area Index to Plant total Biomass production (LAI/BP) to various factors in woody–dominated regions.

**Supplementary Fig. 16.** Attribution of trends in (a) leaf carbon allocation (*η*(leaf)), (c) ratio of Leaf Mass per Area to Leaf Life Cycle (LMA/LC), (e) ratio of Leaf Area Index to Plant total Biomass production (LAI/BP) to various factors in nonwoody–dominated regions.

**Supplementary Fig. 17.** The global distribution of perennial grassland and seasonal dormant grassland.

**Supplementary Fig. 18.** Geographical distribution of the 483 sites included in this analysis.

**Supplementary Table 1.** Definitions and expressions/values of parameters used in our leaf carbon allocation framework. Parameters are listed for the leaf area dynamics prediction module (Cai et al., 2025; Zhou et al., 2025) and leaf economics prediction module (Wang et al., 2023) separately. **AI**: the ratio of mean annual potential evapotranspiration to mean annual precipitation calculated using climate data for a 20–year period. ***b***: A constant value, equalling 0.604169. **GSL**: The length of the continuous above 0℃ period longer than five days. **LAI_max_**: The seasonal maximum LAI. **A_0sum_**: The annual total potential GPP. **fAPAR_max_**: The seasonal maximum fAPAR.

| Parameter | Definition | Values/Expressions | Variability with time | Variability with space | Datasets used for parameter estimation |
| --- | --- | --- | --- | --- | --- |
| Leaf area dynamics prediction module (Cai et al., 2025; Zhou et al., 2025) | | | | |  |
| *f_0_* | The ratio of precipitation to transpiration (dimensionless) | $f_{0}=0.65 \times e^{-b\times{ln}^{2}(\frac{AI}{1.9})}$ | Yes | Yes | MODIS satellite data (Li et al., 2023) |
| *z* | The unit cost of constructing and maintaining leaves | 12.227 mol m^–2^ a^–1^ | No | No | MODIS satellite data (Li et al., 2023) |
| *σ* | The extent to which seasonal LAI dynamics depart from a “square wave” whereby maximum LAI would be maintained over the whole growing season (dimensionless) | 0.771 | No | No | See parameter *m* |
| *m* | The ratio of steady–state LAI to steady–state GPP | $n =(\sigma\times GSL \times\mathrm{LAI}_{max})/ (A_{0sum}\times\mathrm{fAPAR}_{max})$ | Yes | Yes | *A_0sum_*: FLUXNET 2015 and ONEFLUX dataset (Pastorello et al., 2020)  LAI_max_, fAPAR_max_: MODIS (Li et al., 2023) |
| *α* | The constant smoothing factor | 0.067 | No | No | FLUXNET 2015 and ONEFLUX dataset (Pastorello et al., 2020) |
| *k* | Light extinction coefficient | 0.5 | No | No | (Swinehart, 1962) |
| Leaf economics predication module (Wang et al., 2023) | | | | |  |
| *u* | A constant that relates the ageing rate to LMA and photosynthetic capacity | 768 ± 71 | No | No | (Xu et al., 2017) |
| *C_c_* | A multiplier for the plant–level total carbon costs of leaf construction, including all required investments in constructing and maintaining supporting tissues | 6.31 gC gC^–1^ | No | No | Glopnet dataset (Wright & Reich, 2014) |
| *n* | A constant used to convert g C to g biomass | 2.5 g biomass g C^–1^ | No | No | None |
| *a* | The exponent of AI in the aridity–corrected expressions for LMA/LC | 1.14 | No | No | Glopnet dataset (Wright & Reich, 2014)  CPTD V2 dataset (Wang et al., 2022) |

**Supplementary Table 2.** The selection of key factors that affect leaf carbon allocation using forward stepwise regression. All variables except growing season temperature (Gst) were log–transformed. Mean temperature during the growing season (Gst); total photosynthetic photon flux density during the growing season (*I_abs_*); growing season length (*f*); and the aridity index (AI, defined as the ratio of mean annual potential evapotranspiration to precipitation); forest age (age).

| Responses variables | Selected key factors (* if significant) | R^2^ | AIC | BIC |
| --- | --- | --- | --- | --- |
| Deciduous | Gst* | 0.02 | 195.84 | 204.30 |
|  | **Gst* + AI*** | **0.10** | 186.75 | 198.03 |
|  | Gst* + AI* + PPFD | 0.14 | **176.39** | **190.07** |
|  | Gst* + AI* + PPFD + f | 0.15 | 177.44 | 193.86 |
|  | Gst* + AI* + PPFD + f + age | 0.16 | 179.21 | 198.36 |
| Evergreen | Gst* | 0.15 | 444.77 | 456.31 |
|  | **Gst* + AI*** | **0.17** | 436.59 | 451.98 |
|  | Gst* + AI* + PPFD | 0.16 | **354.21** | **372.24** |
|  | Gst* + AI* + PPFD + f | 0.17 | 353.66 | 375.30 |
|  | Gst* + AI* + PPFD + f + age* | 0.18 | 351.21 | 376.45 |

**Supplementary Table 3.** Summary statistics from the regression of observed and predicted leaf carbon allocation against bioclimatic variables for woody plants and nonwoody plants separately. **Gst,** Growing season temperature; **f**, Growing season length; **AI**, the ratio of mean annual potential evapotranspiration to mean annual precipitation.

| Vegetation |  | Gst | p value | ln AI | p value | R^2^ | Number |
| --- | --- | --- | --- | --- | --- | --- | --- |
| All woody species | Observed | 0.021 ± 0.011 | <0.001 | 0.320 ± 0.073 | <0.001 | 0.12 | 418 |
| Deciduous woody |  | 0.022 ± 0.003 | <0.05 | 0.205 ± 0.191 | 0.67 | 0.04 | 88 |
| Evergreen woody |  | 0.020 ± 0.004 | <0.001 | 0.182 ± 0.065 | <0.001 | 0.15 | 330 |
| All grassland |  | 0.025 ± 0.013 | <0.001 | 0.410 ± 0.111 | <0.001 | 0.15 | 84 |
| Seasonal grassland |  | 0.031 ± 0.013 | <0.05 | 0.631 ± 0.155 | <0.001 | 0.27 | 57 |
| Perennial grassland |  | –0.015 ± 0.014 | 0.60 | –0.058 ± 0.140 | 0.71 | 0.02 | 27 |
| All woody species | Predicted | 0.016 ± 0.010 | <0.001 | 0.433 ± 0.032 | <0.001 | 0.48 | 418 |
| Deciduous woody |  | 0.004 ± 0.002 | <0.001 | 0.361 ± 0.071 | <0.001 | 0.67 | 88 |
| Evergreen woody |  | 0.013 ± 0.006 | <0.001 | 0.341 ± 0.034 | <0.001 | 0.74 | 330 |
| All grassland |  | 0.013 ± 0.011 | <0.05 | 0.577 ± 0.112 | <0.001 | 0.67 | 84 |
| Seasonal grassland |  | 0.013 ± 0.015 | 0.311 | 0.521 ± 0.103 | <0.001 | 0.37 | 57 |
| Perennial grassland |  | –0.010 ± 0.001 | <0.001 | 0.482 ± 0.042 | <0.001 | 0.95 | 27 |

**Supplementary Fig. 1.** Workflow of the Leaf Carbon Allocation framework. **Notation**: *A_0_* is the leaf’s initial photosynthetic rate (g C m^–2^ day^–1^); *h_T_* is the Arrhenius function relating *V_cmax_* to temperature (T), equal to unity when T = 25°C; and *m_c_* is the ratio of the Rubisco–limited photosynthetic rate to *V_cmax_*, which is a function of the intercellular CO_2_ partial pressure, the photorespiratory compensation point, and the affinity of Rubisco for CO_2_ versus O_2；_ fAPAR is the fraction of absorbed photosynthetically active radiation; LMA is leaf mass per area (g biomass m^–2^); LL is leaf lifespan (days); GPP is gross primary production (g C m^–2^ day^–1^); BPE is biomass production efficiency (percent); BP is plant total biomass production (g C m^–2^ day^–1^); LAI_max_ is seasonal maximum Leaf Area Index (m^2^ m^–2^); η(leaf)_eve_ is the fraction of evergreen’ biomass allocated to leaves each year; η(leaf)_dec_ is the fraction of deciduous’ biomass allocated to leaves each year.

**Supplementary Fig. 2.** Regression of the ratio of LMA to 365 days residuals (log–transformed Observed LMA/365 – log–transformed Predicted LMA/365) for **(a)** deciduous plants, and the ratio of Leaf mass per area (LMA) to Leaf lifespan (LL) residuals (log–transformed Observed LMA/LL – log–transformed Predicted LMA/LL) for **(b)** evergreen plants against ln AI (the ratio of mean annual potential evapotranspiration to mean annual precipitation) at the individual plant level. Red lines are the regression lines are based on the simple linear regression model; blue lines are based on the mixed effects model with site as a random effect.

**Supplementary Fig. 3.** Linear regressions between observed log–transformed ratio of leaf mass per area (LMA) to 365 days and predicted log–transformed LMA/365 for **(a, c)** deciduous plants, and observed log–transformed ratio of LMA to leaf lifespan (LL) and predicted log–transformed LMA/LL for **(b, d)** evergreen plants, after considering the aridity constraint on LMA and LL. **(a)** and **(b)** are based on the individual plant level and **(c)** and **(d)** are based on site–mean values. The grey lines are 1:1 lines. Red lines indicate that the aridity constraints on LMA and LL were considered based on the coefficients from simple linear regression models fitted to LMA and LL residuals against Aridity Index (AI) (the ratio of mean annual potential evapotranspiration to mean annual precipitation). Blue lines indicate that the aridity constraints on LMA and LL were considered based on the coefficients from the mixed effects model.

**Supplementary Fig. 4.** Linear regressions of (a) observed and predicted ratios of leaf mass per area to leaf lifespan (LMA/LC), and (b) their bias (LMA/LC_pre–obs_), against seasonal maximum leaf area index (LAI_max_). Red line: y=0.

**Supplementary Fig. 5**. Predicted (red) and observed (blue) distributions of the fraction of biomass production allocated to leaves *η*(leaf), for **(a)** seasonally senescent plants (deciduous woody and seasonally dormant non–woody species) and **(b)** persistent perennial plants (evergreen woody and persistent perennial non–woody species).

**Supplementary Fig. 6.** The spatial distribution of residuals between predicted and measured fraction of biomass allocated to leaves. Measured leaf carbon allocation data come from Peng et al. (2023) and Lu et al. (2025). η(leaf) means the fraction of biomass allocated to leaves each year.

**Supplementary Fig. 7. (a)**–**(d)** Partial residual plots for the log–transformed ratio of Leaf Mass per Area (LMA) to Leaf Cycle (LC) against environmental variables. **(e)**–**(h)** Partial residual plots for the log–transformed Leaf Area Index (LAI) against environmental variables. **(i)–(l)** Partial residual plots for the log–transformed biomass production (BP) against environmental variables. **(a)**–**(b), (e)**–**(f) and (i)–(j)** are for seasonally senescent plants (include deciduous woody species and seasonal dominant nonwoody species); **(c)**–**(d), (g)**–**(h)** and **(k)–(l)** are for are for persistent perennial plants (include evergreen woody species and perennial nonwoody species). Predicted: red. Fitted: black, with 95% confidence intervals. **Gst,** Growing season temperature; **AI**, Aridity Index, the ratio of mean annual potential evapotranspiration to mean annual precipitation.

**Supplementary Fig. 8.** Changes in the response of leaf carbon allocation to elevated CO₂ along the aridity index (AI) for **(a)** woody–dominated regions and **(b)** nonwoody–dominated regions on a global scale.

**Supplementary Fig. 9.** Global maps of the predicted fraction of biomass allocated to leaves in 2010 based on different input LAI/fAPAR datasets and different land cover datasets. (a) and (c): Input LAI/fAPAR timeseries from a global prognostic global LAI model (Zhou et al., 2025); (b) and (d): Input LAI/fAPAR timeseries come from MODIS LAI products (Li et al., 2023); (a) and (b): The ESA CCI–LC land cover dataset (Defourny et al., 2023) was used to distinguish evergreen and deciduous dominated regions; (c) and (d): An semi–empirical land cover dataset was used to distinguish evergreen and deciduous dominated regions. The insert histogram showed the distribution of predicted leaf carbon allocation. Areas dominated by croplands snow/ice, and non–vegetated areas are shown in white.

**Supplementary Fig. 10.** Geographical distribution of the 145 elevated CO_2_ experiments included in the analysis. Colours indicate the variables affected by elevated CO_2_ and sizes indicate the number of experiments at the same location to avoid overlap. The dataset come from the MESI dataset (Van Sundert et al., 2023); only experimental data on the effects of increasing carbon dioxide concentration on leaf BP and plant BP were retained. **BP,** plant total biomass production; **Leaf BP**, Leaf biomass production.

**Supplementary Fig. 11.** Trends of predicted global mean leaf carbon allocation based on MODIS LAI/fAPAR (Li et al., 2023) as inputs, and simulated LAI/fAPAR from a prognostic LAI model (Zhou et al., 2025)as inputs. **(a**) is at global scale, **(b)** is based on woody–dominated regions, **(c)** is based on nonwoody–dominated regions. *η*(leaf) means the fraction of biomass allocated to leaves each year.

**Supplementary Fig. 12.** Trends of **(a)** annual mean temperature (K), **(b)** Carbon dioxide concentration (CO_2_, ppm), **(c)** annual total precipitation (mm year^–1^), **(d)** annual mean vapour pressure deficit (VPD, pa), and **(e)** soil moisture from 1901 to 2019 on a global level. Black dashed lines are the regression lines of variables to year across all time ranges.

**Supplementary Fig. 13.** Trends of **(a)** annual mean temperature (K), **(b)** annual total precipitation (mm year^–1^), **(c)** annual mean vapour pressure deficit (VPD, pa), and **(d)** soil moisture from 1901 to 2019 for woody–dominated regions. Black dashed lines are the regression lines of variables to year across all time ranges.

**Supplementary Fig. 14.** Trends of **(a)** annual mean temperature (K), **(b)** annual total precipitation (mm year^–1^), **(c)** annual mean Vapour pressure deficit (VPD, pa), and **(d)** soil moisture from 1901 to 2019 for nonwoody–dominated regions. Black dashed lines are the regression lines of variables to year across all time ranges.

**Supplementary Fig. 15.** Global attribution of predicted trends (Theil–Sen slope coefficients) in **(a)** leaf carbon allocation (η(leaf)), **(c)** the ratio of leaf mass per area to leaf life cycle (LMA/LC), **(e)** seasonal maximum leaf area index (LAI), and **(g)** plant total biomass production (BP), to atmospheric carbon dioxide (CO_2_), photosynthetic photon flux density (PPFD), precipitation (Prec), soil moisture (soilm), temperature (Temp) and vapour pressure deficit (VPD) in woody–dominated regions. **(b)**, **(d),** **(f)** and **(h)** show the contribution of each factor for each time range. Blue dashed lines are the regression lines fitted for each time range, red solid lines are fitted across the full time range from 1901 to 2019. All trends are quantified by Mann–Kendall coefficients.

**Supplementary Fig. 16.** Global attribution of predicted trends (Theil–Sen slope coefficients) in **(a)** leaf carbon allocation (η(leaf)), **(c)** the ratio of leaf mass per area to leaf life cycle (LMA/LC), **(e)** seasonal maximum leaf area index (LAI), and **(g)** plant total biomass production (BP), to atmospheric carbon dioxide (CO_2_), photosynthetic photon flux density (PPFD), precipitation (Prec), soil moisture (soilm), temperature (Temp) and vapour pressure deficit (VPD) in nonwoody–dominated regions. **(b)**, **(d),** **(f)** and **(h)** show the contribution of each factor for each time range. Blue dashed lines are the regression lines fitted for each time range; red solid lines are fitted across the full time range from 1901 to 2019. All trends were quantified by Mann–Kendall coefficients.

**Supplementary Fig. 17.** The global distributions of perennial and seasonally dormant grassland. Perennial grasslands (maintaining green foliage year–round) and seasonally dormant grasslands (undergoing dormancy or shedding) were classified according to the following steps. First, we determined the growing season length (GSL) for each grassland site using smoothed LAI data. The growing season was identified with the bigleaf package (Knauer et al., 2018), which defines vegetation as active when its LAI exceeds 5% of the annual peak (95th percentile). Then areas with the GSL larger than 0.95 were classified as perennial grassland, while those below were considered seasonally dormant.

**Supplementary Fig. 18.** Geographical distribution of the 483 sites included in this analysis. These site–level measurements come from Peng et al. (2023) and Lu et al. (2025). Seasonally senescent plants include deciduous woody species and seasonal dominant nonwoody species; persistent perennial plants include evergreen woody species and perennial nonwoody species.

**References**

Cai, W., Zhu, Z., Harrison, S. P., Ryu, Y., Wang, H., Zhou, B., & Prentice, I. C. (2025). A unifying principle for global greenness patterns and trends. *Communications Earth & Environment*, *6*(1), 19. https://doi.org/10.1038/s43247–025–01992–0

Knauer, J., El–Madany, T. S., Zaehle, S., & Migliavacca, M. (2018). Bigleaf—An R package for the calculation of physical and physiological ecosystem properties from eddy covariance data. *PLOS ONE*, *13*(8), e0201114. https://doi.org/10.1371/journal.pone.0201114

Li, B., Ryu, Y., Jiang, C., Dechant, B., Liu, J., Yan, Y., & Li, X. (2023). BESSv2.0: A satellite–based and coupled–process model for quantifying long–term global land–atmosphere fluxes. *Remote Sensing of Environment*, *295*, 113696. https://doi.org/10.1016/j.rse.2023.113696

Lu, X., Chung, C.–H., Malhi, Y., & Huang, C. (2025). Global Patterns in the Allocation of Forest Net Primary Production. *Journal of Biogeography*, *52*(4), e15094. https://doi.org/10.1111/jbi.15094

Pastorello, G., Trotta, C., Canfora, E., Chu, H., Christianson, D., Cheah, Y.–W., Poindexter, C., Chen, J., Elbashandy, A., Humphrey, M., Isaac, P., Polidori, D., Reichstein, M., Ribeca, A., van Ingen, C., Vuichard, N., Zhang, L., Amiro, B., Ammann, C., … Papale, D. (2020). The FLUXNET2015 dataset and the ONEFlux processing pipeline for eddy covariance data. *Scientific Data*, *7*(1), Article 1. https://doi.org/10.1038/s41597–020–0534–3

Peng, Y., Prentice, I. C., Bloomfield, K. J., Campioli, M., Guo, Z., Sun, Y., Tian, D., Wang, X., Vicca, S., & Stocker, B. D. (2023). Global terrestrial nitrogen uptake and nitrogen use efficiency. *Journal of Ecology*, *111*(12), 2676–2693. https://doi.org/10.1111/1365–2745.14208

Swinehart, D. F. (1962). The Beer–Lambert Law. *Journal of Chemical Education*, *39*(7), 333. https://doi.org/10.1021/ed039p333

Van Sundert, K., Leuzinger, S., Bader, M. K.–F., Chang, S. X., De Kauwe, M. G., Dukes, J. S., Langley, J. A., Ma, Z., Mariën, B., Reynaert, S., Ru, J., Song, J., Stocker, B., Terrer, C., Thoresen, J., Vanuytrecht, E., Wan, S., Yue, K., & Vicca, S. (2023). When things get MESI: The Manipulation Experiments Synthesis Initiative—A coordinated effort to synthesize terrestrial global change experiments. *Global Change Biology*, *29*(7), 1922–1938. https://doi.org/10.1111/gcb.16585

Wang, H., Harrison, S. P., Li, M., Prentice, I. C., Qiao, S., Wang, R., Xu, H., Mengoli, G., Peng, Y., & Yang, Y. (2022). The China plant trait database version 2. *Scientific Data*, *9*(1), 1–13. https://doi.org/10.1038/s41597–022–01884–4

Wang, H., Prentice, I. C., Wright, I. J., Warton, D. I., Qiao, S., Xu, X., Zhou, J., Kikuzawa, K., & Stenseth, N. Chr. (2023). Leaf economics fundamentals explained by optimality principles. *Science Advances*, *9*(3), eadd5667. https://doi.org/10.1126/sciadv.add5667

Wright, I., & Reich, P. (NaN). *GLOPNET – Global Plant Trait Network Database*. https://doi.org/10.5063/AA/farshid25.15.2

Xu, X., Medvigy, D., Joseph Wright, S., Kitajima, K., Wu, J., Albert, L. P., Martins, G. A., Saleska, S. R., & Pacala, S. W. (2017). Variations of leaf longevity in tropical moist forests predicted by a trait–driven carbon optimality model. *Ecology Letters*, *20*(9), 1097–1106. https://doi.org/10.1111/ele.12804

Zhou, B., Cai, W., Zhu, Z., Wang, H., Harrison, S. P., & Prentice, I. C. (2025). A General Model for the Seasonal to Decadal Dynamics of Leaf Area. *Global Change Biology*, *31*(3), e70125. https://doi.org/10.1111/gcb.70125
